# A Comprehensive Framework for Measuring the Immediate Impact of TV Advertisements: TV-Impact

**DOI:** 10.3390/e26020109

**Published:** 2024-01-25

**Authors:** Afra Arslan, Koray Tecimer, Hacer Turgut, Ömür Bali, Arda Yücel, Gülfem Isiklar Alptekin, Günce Keziban Orman

**Affiliations:** 1 Research and Development Center, iLab, 34736 Istanbul, Turkey; aarslan@ilab.com.tr (A.A.); ktecimer@ilab.com.tr (K.T.); obali@ilab.com.tr (Ö.B.); ayucel@ilab.com.tr (A.Y.); 2 Computer Engineering Department, Galatasaray University, 34349 Istanbul, Turkey; gisiklar@gsu.edu.tr (G.I.A.); korman@gsu.edu.tr (G.K.O.)

**Keywords:** television advertising, causal inference, counterfactual, TV-ad impact measurement, web session traffic, supervised learning, machine learning

## Abstract

Measuring the immediate impact of television advertisements (TV ads) on online traffic poses significant challenges in many aspects. Nonetheless, a comprehensive consideration is essential to fully grasp consumer reactions to TV ads. So far, the measurement of this effect has not been studied to a large extent. Existing studies have either determined how a specific focus group, i.e., toddlers, people of a certain age group, etc., react to ads via simple statistical tests using a case study approach or have examined the effects of advertising with simple regression models. This study introduces a comprehensive framework called TV-Impact. The framework uses a Bayesian structural time-series model called CausalImpact. There are additional novel approaches developed within the framework. One of the novelties of TV-Impact lies in its dynamic algorithm for selecting control variables which are supporting data sources and presumed to be unaffected by TV ads. In addition, we proposed the concept of Group Ads to combine overlapping ads into a single ad structure. Then, Random Forest Regressor, which is a commonly preferred supervised learning method, is used to decompose the impact into single ads. The TV-Impact framework was applied to the data of iLab, a venture company in Turkey, and manages its companies’ advertising strategies. The findings reveal that the TV-Impact model positively influenced the companies’ strategies for allocating their TV advertisement budgets and increased the amount of traffic driven to company websites, serving as an effective decision support system.

## 1. Introduction

Throughout history, mass media has served as a powerful tool for marketers to capture consumer attention and promote their products. Television (TV) advertising, in particular, has consistently been the preferred medium for reaching a wide audience. According to Statista (2023), global spending on TV and video advertising is projected to reach 326.2 billion USD in 2023, which still represents approximately 30% of all advertising expenditures across all channels in that year. In Turkey, where the growth rate of digital advertising is lower but the ratio is close to 50%, this makes the analyses of TV-ad impact on online traffic more valuable [1].

Consumer attitudes towards television advertisements (TV ads) have evolved over the years due to shifts in media consumption habits, technological advancements, and changing advertising strategies [2]. As a result, understanding the impact of TV ad campaigns has been a crucial endeavor for companies looking to optimize their return on investment (ROI) in advertising expenditures and make efficient data-driven decisions. The effective measurement of TV ads allows advertisers to identify which advertisements successfully reach their target audiences, enhance brand awareness, boost sales, and yield a positive ROI [3]. This measurement, however, is complex due to the influence of various non-advertising factors, such as daily life dynamics, social and humanitarian factors, seasonality effects, etc. Although the exact measurement of TV ads seems analytically complicated, we can still profit from data analysis approaches.

### 1.1. Motivation

The challenge in measuring TV-ad impact lies in TV being an offline medium and the presence of numerous non-advertising factors affecting online traffic. Our study aims to isolate the impact of TV ads on online traffic, focusing on differentiating the effects of various ads while minimizing bias. Additionally, investigating the immediate effects of ads in the study may increase the likelihood of macroeconomic factors in volatile markets or other environmental indicators having similar effects over short time intervals.

We introduce the TV-Impact framework, which examines the causal relationship between TV ads and a company’s online traffic. This framework is tested using data from 11 diverse companies under iLab. These companies are all part of iLab https://www.ilab.com.tr/en/ (accessed on 18 January 2024), one of Turkey’s leading advertisers, renowned for its prominent presence in the country’s digital ecosystem. Collectively, iLab’s group companies reach 65% of the Turkish internet audience and employ over 2000 people. iLab’s advertising strategies involve a comprehensive approach, including social media analysis, marketing mix modeling, and brand tracking tools.

Our research aims to measure the individual impact of a company’s TV ad, distinguishing it from others. The base model used in the TV-Impact framework is the CausalImpact, the causal inference model proposed by Google [4]. The CausalImpact model uses Bayesian structural time series (BSTS) to predict the effects of interventions through counterfactual scenarios based on control variables. The BSTS models have been extensively applied for diverse purposes such as exploring the association between Bitcoin’s market price and economic factors [5], examining the demographic heterogeneity and time variation in the vaccine effect on COVID-19 propagation [6], investigating the impacts of the long-standing Taliban insurgency [7], and analyzing cannibalization effects due to individual promotions [8].

### 1.2. Contributions

Most of the previous works dedicated to TV-ad impact measurement were performed from the perspectives of the social sciences [9,10,11]. Although they develop quantitative analysis, it is based on simple statistical tests of the data of real humans obtained via surveys. Our paper contributes significantly to the field by proposing a comprehensive framework to assess individual TV-ad impacts on online platforms, a first in this domain. Other contributions can be listed as follows:

- Development of a generic and comprehensive infrastructure suitable for use by all TV advertisers;
- Provision of detailed descriptions of all necessary data definitions, information flows, algorithm pseudocodes, and measurement approaches in the field of assessing the immediate impact of TV ads;
- Application of CausalImpact on real-life TV-ad data;
- Proposition and comparison of three distinct approaches for measuring the impact of TV ads within the framework;
- Proposition of a novel dynamic control variable selection procedure in the CausalImpact model;
- Separation of the impact of group advertisements (Group Ads) from individual advertisements via the distribution of their cumulative impacts.

The rest of the article is organized as follows: in Section 2, we present a literature survey of the research focusing on investigating the influence of TV ads. In Section 3, we describe and analyze the dataset and detailed definitions of the employed terms throughout the article. Next, the base methodology and our proposed framework are described in Section 4. Section 5 presents the experiments and obtained results, and finally, in Section 6, we conclude the paper.

## 2. Related Work

This section searches through the existing literature on the effects of TV ads. The work of Lodish et al. is one of the analyses on traditional ad effectiveness [12], revealing that while increased budgets do not guarantee higher sales, changes in brand, copy, and media strategies could be beneficial. A subsequent study concentrated on the infomercial ad genre, assessing its distinct effects on sales [13]. The authors surveyed 878 participants and identified that factors like product endorsements, celebrity endorsements, and product comparisons influenced purchasing behaviors based on consumer age. However, the study was limited on a single type of TV ad.

A work on the attention and interest generation created by TV ads is conducted by Ansari and Joloudar [3]. Their findings highlighted the effectiveness of TV ads in capturing attention, sparking interest, creating desire, and driving purchasing action, as evidenced by their control groups. However, they did not explore their varied impacts across digital channels. In their 2011 and 2012 publications, Vaver and Kohler pioneered a different approach to establish control groups based on geography for their experiments [14,15]. They introduced geographic control groups for ad impact measurement and emphasized the need for periodic reevaluation of ad effects.

In 2014, Kitts et al. investigated the lagged effects of TV ads, noting how they induce spikes in web traffic and keyword searches after a specific duration [16]. Their findings demonstrate the immediate influence of TV ads on digital traffic. This immediate impact constitutes a crucial component of our research. This study also introduces the first time the term group advertisements refer to ads broadcast concurrently across various TV channels. Their terminology facilitated the definition of the aggregated assessment of their cumulative impacts.

In their 2014 study, Joo et al. emphasized the infrequent coordination between TV ads and digital search ad campaigns despite the increasing prevalence of integrated marketing practices [17]. Their research found that user behaviors in the digital realm could shape TV ad campaigns, even as integrated marketing became more widespread. Additionally, they analyzed the impact on click-through rates in addition to the search frequency.

Lewis and Rao discussed challenges in assessing advertising campaign effectiveness, especially in controlled trials [18]. The authors emphasized the often prohibitive expenditures and infeasibility of ad experiments for numerous companies, accentuating the difficulties when field experiments are tied to individualized sales metrics.

A similar paper to ours enhances the existing research on the interplay between different media by examining the relationship between TV advertising and online shopping behaviors [19]. The study confirms a direct link between TV advertising and increased online shopping, and it also examines how different factors, such as the advertisement’s content and where it is placed in the media, can affect this relationship.

Tirunillai and Tellis assessed TV ads’ impact on online discussions, analyzing short-term and long-term effects [20]. Carreon et al. evaluated the influence of ad exposure duration on purchasing behaviors, finding that demographic information plays a significant role [21]. Their findings revealed that a model incorporating both users’ demographic information and ad exposure did not significantly outperform a model composed solely of demographic information.

Sinha, Saini, and Arbour made a noteworthy contribution to the field by conducting a study to predict treatment effects with precision, leveraging the creation of multiple control groups [22]. However, their study did not incorporate feature extraction during non-ad periods and did not address the topic of group ads.

Our study builds on these findings, addressing gaps such as the detailed impact of TV ads on digital traffic and the distribution of cumulative impacts of group advertisements. We introduce the “TV-Impact” framework, offering a comprehensive approach to understanding the evolving connection between traditional advertising and the digitalized user experience.

## 3. Dataset

The overall datasets that we have used are from iLab. It incorporates data from 11 distinct companies, each representing varied sectors and employing different advertising strategies. For each company, two primary data sources are utilized: *i. Online traffic data*, collected from the respective company’s website; *ii. TV-ad data*, sourced from the associated advertising agency.

### 3.1. Online Traffic Data

Online traffic logs are collected using the Google Analytics tool https://analytics.google.com/analytics/ (accessed on 18 January 2024). This tool captures session information for users visiting the site. On average, 60,000 sessions are recorded daily for each company. These data are aggregated from both the company’s website and their mobile application, if any. It shows the number of instant sessions collected on the platform. The sessions are categorized based on their origin: (i) desktop and (ii) mobile. While desktop sessions are sub-categorized as direct, organic, paid, and referral, mobile ones are sub-categorized as Android and iOS, which reflect the source platform.

Direct sessions represent users who access the website by directly entering the URL or using a bookmark. Organic sessions encompass those who discover the site through unpaid search engine results. Paid session traffic comprises visitors who reach the site by clicking on sponsored advertisements. The referral one comes from users clicking hyperlinks in external sources like blogs, news articles, social media, or partner websites. Collaborative efforts and content sharing can affect it. Our framework prioritizes these four key session types due to their prevalence, with the flexibility to introduce additional types as needed. A sample of online traffic logs is represented in Table 1.

For every single company, we prepare a dedicated dataset by using the online traffic logs. As the data preparation step, we process the following steps.

- Sessions are grouped in 10 s intervals to reduce the excess zeros in the session data.
- We calculate statistical measures, including the mean, median, and quartile values, based on traffic data from the last 7, 15, 30, and 60 days, to ensure robust data analysis. Shorter time intervals enable us to gauge current trends, while longer durations help us capture seasonality. This approach of utilizing calculated statistics rather than raw time-series data are imperative for minimizing the impact of outliers and accurately discerning trends.
- Before the statistical analysis, we excluded time intervals corresponding to the company’s TV ads so that the derived statistics represent periods not immediately influenced by TV ads. These time periods are marked as −1 in the data.

As a result of these stages, we obtain statistically enriched datasets for each company under consideration. The data preparation procedure is run for newly arrived data on a daily basis. Since there are no null values in the data, there is no need for an imputation step. Anomaly control is performed by data teams outside the framework, so raw traffic data are considered clean. At the end, these datasets encapsulate 63 features for each 10 s time interval, as depicted in Table 2.

In the proposed framework, online traffic data from other companies are used as auxiliary data for the company under consideration. To ensure the integrity of these auxiliary company data, we make sure to use data from time periods, when there were no ad effects. Henceforth, the term online traffic data will denote the dataset containing all companies’ enriched statistical data.

### 3.2. TV-Ad Data

The secondary data source encompasses advertising data, systematically aggregated daily from the collaborative advertising agency. Given the variability in advertising strategies and duration across companies, the average daily ad count per company fluctuates. The dataset, which focuses on TV ads, includes 11 features such as broadcast date, time, duration, channel, and associated program as outlined in Table 3.

Notably, sequential TV ads may be broadcast in close succession, leading to potential residual effects from preceding ads. This temporal overlap complicates the differentiation of the effects of TV ads that have been broadcast in the same or a very close time frame. To address this issue, our framework groups these ads and treats them as a single, very long ad named as a Group Ad. In contrast, commercials without temporal overlap with other ads are classified as Individual Ads.

**Figure 1:**
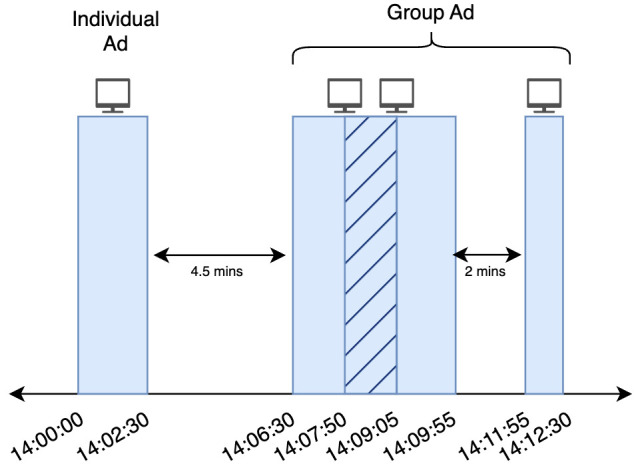
Illustration of an Individual Ad and a Group Ad where impact duration is set to 4 min.

This grouping is facilitated by defining an advertising impact duration parameter, denoted as *t*. For each advertisement, we analyze the time interval beginning at the advertisement’s start and the extending *t* minutes beyond its conclusion. When we examine these time intervals, if intersecting TV ad groups are formed, these ads are classified as a Group Ad. Figure 1 provides an illustrative example where *t* is set at 4 min and four consecutive ads are broadcast. The first ad, with no subsequent ad broadcast within 4 min of its conclusion, is categorized as an Individual Ad. The following three ads, however, share overlapping 4 m impact durations and are thus grouped as a Group Ad.

## 4. Methodology

This section will discuss the causal inference analysis and the methods and parameters used in the proposed framework.

### 4.1. Causal Inference Analysis

The framework presented in this paper centers on the application of causal inference, which seeks to elucidate the causal relationship between an intervention (event) and its resulting effect. The effect of an intervention is measured by predicting a counterfactual and comparing it with the actual outcome. The counterfactual is a hypothetical scenario or state of affairs that represents what could have happened or what the outcome might have been if a specific event, action, or intervention had not occurred [4,23,24]. The temporal context is segmented into pre- and post-intervention periods. Basically, pre-period information is modeled to predict post-period as the counterfactual. The counterfactual is then compared with the actual to estimate intervention effect. CausalImpact is a causal inference model developed by Google [4]. In this study, Google shows how Bayesian structured time-series models are state-space models governed by the following mathematical equations:

(1) $$\begin{array}{c} y_{t}=Z_{t}^{T}\alpha_{t}+\varepsilon_{t} \end{array}$$

(2) $$\begin{array}{c} \alpha_{t+1}=T_{t}\alpha_{t}+R_{t}\eta_{t} \end{array}$$

The initial equation establishes a linkage between the observation $y_{t}$ and the state vector $\alpha_{t}$, incorporating the observation error $\varepsilon_{t}$ and the output matrix $Z_{t}^{T}$. The subsequent expression represents the state equation, establishing a connection between the state $\alpha_{t}$ at time *t* and the subsequent state $\alpha_{t+1}$ at t+1 via the employment of the transition matrix $T_{t}$ and $R_{t}$, denoted as $\eta_{t}$, serving as a variable to integrate state components, such as seasonality and trend. To derive a posterior distribution for states, Markov Chain Monte Carlo (MCMC) sampling techniques are employed. CausalImpact employs control variables to estimate the counterfactual. Control variables should be correlated with the target variable but remain unaffected by the intervention. The model learns the relationships between these control variables and the target variable during the pre-period. With this acquired knowledge, the model can estimate the time-series behavior of the target variable during the post-period.

Brodersen et al. experimented with their model of an ad campaign as an intervention, with the cumulative count of organic and paid clicks serving as the target metrics over weeks-long pre- and post-periods [4]. While this approach is insightful, it encounters limitations in the form of external distortions over extended periods. That is why, differently from them, we concentrated on the immediate impact. Our research diverges primarily in the selection of pre- and post-intervention intervals. We focus on the immediate impact of TV advertisements, scrutinizing the effects over shorter intervals, such as minutes. This granularity is critical in our context, considering the variability in advertising impact based on factors like time of day, channel, and program content. Another reason for focusing on short-term impact is that we need data that is not affected by any TV ad for counterfactual calculation. Longer time frames pose challenges, as they are more likely to encompass multiple ads.

### 4.2. Calculating Immediate TV-Ad Impact

**Figure 2:**
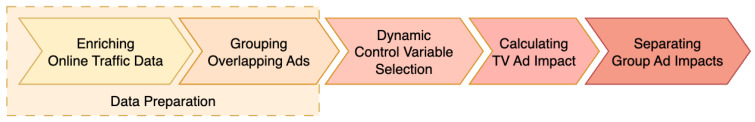
Flow of TV-Impact framework.

As illustrated in Figure 2, the TV-Impact framework is structured into four distinct stages. The first stage, designated as the Data Preparation phase, is dedicated to enriching online session data and categorizing ads into Individual and Group Ads, as mentioned in Section 3. This foundational step is crucial for ensuring the quality and relevance of the data underpinning the subsequent analysis.

Subsequent sections will thoroughly examine two components: firstly, the selection of control variables for causal inference modeling, and secondly, the computation of TV ad effects, especially the effects of TV ads within Group Ads.

#### 4.2.1. Dynamic Control Variable Selection

Our dynamic control variable selection process relies on calculating the counterfactual, which is usually based on the pre-period of a single company’s advertisement data source. Relying solely on one data source can result in any anomalies or different influencing factors in the data, directly affecting the prediction. Therefore, it is important to enhance the reliability of predictions by incorporating different data sources. In our proposed TV-Impact framework, we consider several data sources to overcome the mentioned problems.

**Figure 3:**
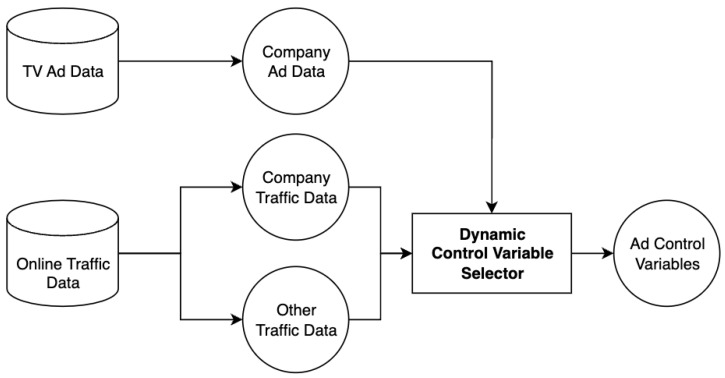
Selection of control variables for a specific company advertisement.

Figure 3 illustrates the flow of the dynamic control variable selection process. A certain number of control variables to be used in predicting the counterfactual for each TV ad are dynamically selected based on correlation analysis. Algorithm 1 details this selection methodology. The function takes the TV-Ad Data *A* and Online Traffic Data *T* as input. It also accepts input parameters, including the correlation threshold (*thr*) and control variable limit (*limit*). The purpose of *thr* is to select control variables that bear a certain level of similarity to the pre-period of the target variable. A high *thr* value can result in selecting none or very few control variables, whereas a low value may lead to an excessive selection. Given the brevity of the pre-period in our problem, selecting too many control variables may lead to the model learning from data noise instead of actual trends. To prevent this, we empirically set a (*limit*) on the number of control variables.
**Algorithm 1:** Dynamic Control Variable Selection  
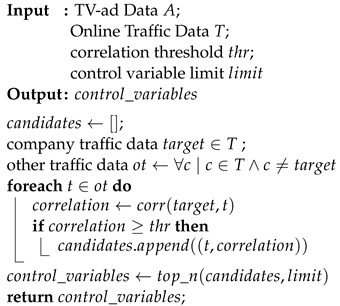


Based on the start and end times of a TV ad learned from *A*, traffic data from the company that broadcast the TV ad (*target*) and the remaining traffic data (*ot*) are determined from dataset *T*. For each traffic data (*t*) in *ot*, the correlation with the target is calculated. If the correlation is greater than or equal to the threshold (*thr*), then *t* is added to the candidates list. This process is repeated for all *t*’s. Following this, the *limit* number of variables with the highest correlation scores in the candidates list are selected as control variables.

#### 4.2.2. Measuring TV-Ad Impact

**Figure 4:**
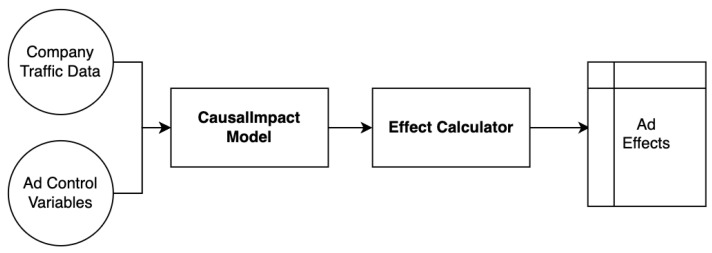
The flow of causal impact calculation of TV ads.

In this section, we explore three distinct methods for quantifying the impact of TV ads (Figure 4). A fundamental approach involves calculating the exact difference between the actual and the counterfactual. However, this straightforward approach presents several inherent problems within the context of our analysis. First, when the estimated counterfactual value is substantially higher than the actual value, we observe a misleading negative impact as a result. In reality, TV ads do not create negative impact on online traffic.

Second, the counterfactual inherently includes minor fluctuations in session counts driven by inherent uncertainties. These non-advertising-related fluctuations can lead to misleading results by calculating non-effectual increases as if they were effects. To overcome the mentioned limitations of the basic approach, we propose three novel alternative extensions: *pos_impact*, *cum_impact*, and *upper_impact*.

*i*.
pos_impact

The first method, pos_impact, involves restricting the consideration of data points to the post-period where only a positive impact is observed. Subsequently, these identified positive impact data points are aggregated to compute the total impact. Equation (3) describes the function that is used to find out the positive difference between the actual and the counterfactual. Equation (4) describes the calculation of pos_impact where *y* is the actual, $\hat{y}$ is the counterfactual, and *n* is the number of data points in the post-period:

(3) $$\begin{array}{c} \delta \left(x,y,z\right)=\left\{\begin{array}{cc} x-y, & \mathrm{if}\;x>z\\ 0, & \mathrm{otherwise} \end{array}\right. \end{array}$$

(4) $$\begin{array}{c} pos\_impact=\sum_{i=1}^{n}\delta \left(y_{i},{\hat{y}}_{i},{\hat{y}}_{i}\right) \end{array}$$

This approach effectively mitigates the problem of negative effects by exclusively aggregating positive data points. Nevertheless, it introduces a potential challenge by accounting for even minor fluctuations as consequential effects. This inclusion of small fluctuations results in an optimistic bias since only positive fluctuations are considered, while negative ones are not considered.

*ii*.
cum_impact

As an alternative, cum_impact entails determining the effect by identifying, initially, the cumulative impact of first *k* post-period data points (5). Then, it calculates the maximum cumulative impacts among each possible k≤n value (6).

(5) $$\begin{array}{c} cum\_impact_{k}=\sum_{i=1}^{k}y_{i}-{\hat{y}}_{i} \end{array}$$

(6) $$\begin{array}{c} cum\_impact=max({\left\{cum\_impact_{i}\right\}}_{i=1}^{n}) \end{array}$$

While this method does indeed address the problem of creating a significant positive bias and resolves the problem of negative impact, it lacks consistency. It relies on cumulative sum calculations of effect changes based on whether the observed negative effect occurs before or after the initial impact of the advertisement. High estimates of the counterfactual prior to the effect can still impact the reliability of our model.

*iii*.
upper_impact

The third approach, upper_impact, involves computing the effect with confidence intervals. The Bayesian model employed in counterfactual estimation produces a posterior distribution for the estimates, which can be used to establish a threshold. Equation (7) uses eqrefeq:delta and calculates upper_impact by taking upper confidence levels (ρ) as the threshold:

(7) $$\begin{array}{c} upper\_impact=\sum_{i=1}^{n}\delta \left(y_{i},{\hat{y}}_{i},\rho_{i}\right) \end{array}$$

#### 4.2.3. Separation of Group Ad Impacts

**Figure 5:**
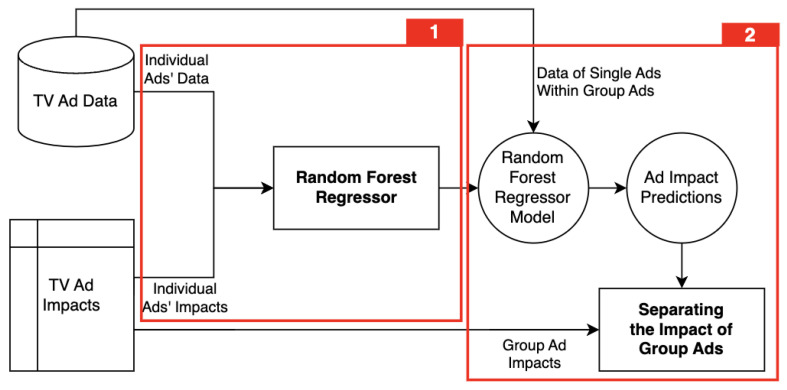
The flow of separating Group Ad impacts for the single ads within Group Ads.

Group Ads are evaluated as a single advertisement during impact calculation. However, in our problem, it is crucial to measure the impact of each TV ad individually. To achieve this, it is necessary to separate the Group Ad impacts into individual impacts. As a solution to this, we employ an artificial learning (AI)-based approach. We represent the schema of our approach in Figure 5. In the first step, a dataset of characteristics and impacts of each Individual Ad is used as the learning set. Here, the impacts are used as the target variable, while the characteristics are the inputs of the AI model. Table 3 shows an example of these characteristics. A Random Forest Regressor (RFR) [25] is trained to learn the impact of each Individual Ad at this step.

**Figure 6:**
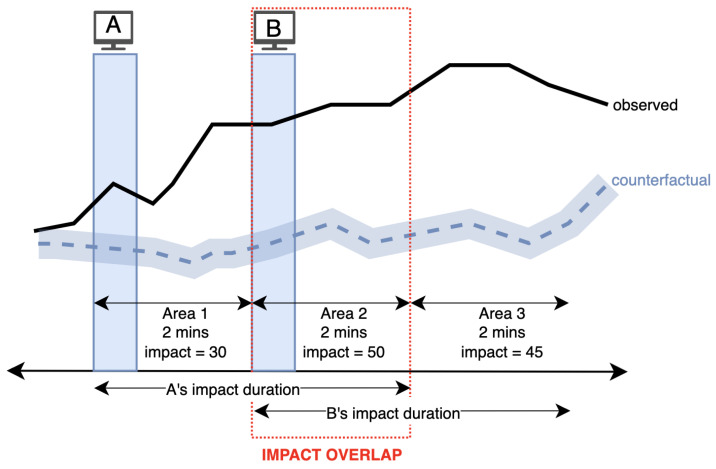
Overlapped impact of two single ads within a Group Ad.

In the second step, the impact of each single Ad, which is a part of a Group Ad, is predicted by the model built in the first step. These predicted impact values are used as coefficients to separate the impact where the impacts of single ads overlap. For example, let us consider a Group Ad consisting of two single ads as illustrated in Figure 6, and the advertising impact duration *t* is set to 4 min. We consider an impact as the number of unique sessions resulting from a TV ad. Since in Area 1 and Area 3, there is no overlapped impact in the Group Ad, calculated impacts in these areas are assigned to single ads in the corresponding areas. In other words, 30 sessions in Area 1 is solely from single ad A, and the 45 sessions in Area 3 is from single ad B. On the other hand, in Area 2, there is an impact overlap. Using the RFR trained in the previous step, the impact of these single ads is predicted and used to separate the impact in proportion to these values. Assuming that their impact predictions are 40 and 60, the actual impact of single ads A and B become 20 and 30, respectively. As a result, the total impact of Group Ad, which is 125, is distributed as 50 from single ad A and 75 from ad B.

## 5. Experiments and Results

### 5.1. Imaginary Ad Data

Evaluating the performance of the TV-Impact framework presents a complex endeavor, primarily due to the absence of verifiable ground-truth data regarding the impacts of TV ads on online sessions. Nevertheless, evaluating time-series forecasting models, which predict the counterfactual, is feasible. As proposed in [4], the CausalImpact model can be evaluated via imaginary interventions. Imaginary intervention data are generated from the target data without intervention during a time frame. For an imaginary intervention, a counterfactual prediction is made as if there was an intervention, and the goal is to make the counterfactual as close as possible to the actual outcome due to the absence of intervention. This methodology facilitates fine tuning the CausalImpact model parameters to minimize error rates in the imaginary intervention data. In our problem, data that does not involve a TV-ad impact in the online session data were identified for this purpose, and these data were referred to as *Imaginary Ad Data*.

The Imaginary Ad Data were extracted from the comprehensive online traffic data of 11 companies involved in the work. While the results across these companies exhibited similarities, this paper focuses on Imaginary Ad data from three companies, selected based on data clarity and volume. These companies are anonymized as Company 1, Company 2, and Company 3. The examined data encompasses a 15-day period, yielding a count of 472, 660, and 507 Imaginary Ads from these companies, respectively. It should be noted that the findings and interpretations discussed herein are applicable across all participating companies.

### 5.2. Parameter Tuning Metrics

Two key error metrics are employed to find optimal framework parameters, *t*, *limit* and *thr*. These are Root Mean Squared Error (RMSE) (8) and Mean Absolute Error (MAE) (9). Here, *y* is the actual, $\hat{y}$ is the counterfactual, and *n* is the number of data points in the post-period.

(8) $$RMSE=\sqrt{\frac{1}{n}\sum_{i=1}^{n}{\left(y_{i}-\hat{y_{i}}\right)}^{2}}$$

(9) $$MAE=\frac{1}{n}\sum_{i=1}^{n}\left|y_{i}-\hat{y_{i}}\right|$$

These error metrics serve the purpose of quantifying the model’s predictive quality and its capacity to capture the salient features of the data in the post-period. In the experiments, framework parameters were selected based on the lowest error scores.

### 5.3. Model Parameters

This section presents the parameters used within the framework. For the CausalImpact model, we experimented with different parameter values for the model and observed that the default values performed the best. Therefore, we stuck with the default settings as specified in the library http://google.github.io/CausalImpact/CausalImpact.html (accessed on 18 January 2024). The parameters pre_period and post_period were aligned with the ad impact duration (*t*) in our study, as determined via experiments in Section 5.3.1. For the implementation of the Random Forest Regressor, the scikit-learn library was used https://scikit-learn.org/stable/modules/generated/sklearn.ensemble.RandomForestRegressor.html (accessed on 18 January 2024). Here, after employing the Grid Search hyperparameter selection method, the parameters n_estimators and max_depth were established as 500 and 15, respectively. The remaining parameters were retained at their default values. Furthermore, the critical framework parameters of the correlation threshold (thr) and the limit on control variables (limit) were determined via a series of experimental evaluations, as detailed in Section 5.3.2.

#### 5.3.1. Advertising Impact Duration (*t*)

To find the optimal value for the *t* parameter, one can test several different *t* values. However, increasing the *t* leads to an increase in the overlap between the consecutive ad’s impact duration, increasing the number of Group Ads. As seen in Table 4, the higher the *t* values, the lower the average number of individual ads generated and the higher the number of ads within Group Ads.

This fact negatively impacts the framework because the decrease in the number of individual ads negatively affects the performance of the model used to separate group ad impacts. Therefore, our experiments tested *t* values of 2, 3, 4, and 6, and higher values were not considered.

For each company in the Imaginary Ad Data, imaginary ads were generated with these different *t* values, followed by predictions for the post-period. Since these are not real ads, the model’s predictions and the actual values were expected to be close to each other. The model prediction error was measured using RMSE and MAE scores. As indicated in Table 5, the *t* of 4 and 6 min revealed the lowest error rates. However, considering the balance between individual and Group Ads as shown in Table 4, a 4 min duration was selected as the optimal ad impact duration for our framework.

#### 5.3.2. Correlation Threshold (thr) and Control Variable Limit (limit)

As Section 4.2.1 explains, control variables used for counterfactual prediction should be correlated with the target. Furthermore, the number of control variables should be limited. Experiments were conducted on the Imaginary Ad Data to determine the thr and limit parameter values. These experiments tested various thr and limit values across three companies.

Since the prediction error obtained by MAE and RMSE represent similar results, here we evaluate the RMSE scores in post-period prediction in detail (Table 6). For Company 1 and 2, a thr of 0.5 yielded the best results, while for Company 3, 0.6 provided the best outcome. In terms of the limit parameter, setting it to 5 optimized the performance for Companies 1 and 3, while the limit of 3 was ideal for Company 2. Based on these findings, the framework was configured to employ a thr parameter value of 0.5 and a limit of 5, thus optimizing the model’s predictive accuracy for the companies evaluated.

### 5.4. Overall Evaluation

After the parameter setting step, we execute our TV-Impact framework to reveal the relationships between the characteristics of ads (channel, program, time, etc.) and their respective impacts. It is important to note that optimizing ad campaigns and negotiating deals are iterative and dynamic processes. New agreements may be forged during an advertising campaign, potentially leading to superpositions among successive ads from the same company. If consecutive ads from different companies have a short duration in between, we solve this problem with our Group Ad concept. However, evaluating the individual effects of advertising decisions can be challenging if the same company’s ads are on screen for different periods.

Moreover, the choice of evaluation metrics, such as the number of online sessions, presents its own set of difficulties. Regardless of which evaluation metric is chosen, it depends on many parameters beyond the scope of advertising. Hence, conducting this evaluation is a completely different task and goes beyond the development of the framework. We do not propose a fully-fledged evaluation system in this work, as that is not our main motivation. Still, we propose to evaluate the TV-Impact framework efficiency by comparing the monthly number of sessions of the current year with the ones of the previous year, thereby allowing for the integration of seasonal variability in the assessment.

Table 7 reveals that Company 1 experienced an average increase of 41.5% in online session numbers compared to the previous year, along with an average decrease of 42.5% in ad expenditure per ad. While the other two companies also observed a decrease in ad spending, their online session numbers decreased. We should remember that during the period under review, an important earthquake disaster occurred in Turkey, potentially exerting substantial effects on the marketing and advertising sectors.

That is why the fact that the decrease in spending is greater than the decrease in session numbers can be considered a positive sign. As previously stated, the increase or decrease in session numbers is not solely attributable to the framework but also includes external, often unquantifiable factors, such as the aforementioned earthquake. Given this and following recommendations from the ad department, we accord greater significance to the “Spending per Ad” metric. A decrease in this metric across all three companies was observed, suggesting the overall efficacy of the TV-Impact framework.

Furthermore, the absence of a comparable benchmark framework in the existing literature makes it impossible to directly compare our framework with others. Nonetheless, our proposed TV-Impact framework effectively quantifies the immediate effects of TV ads on online traffic. This achievement is significant, considering the lack of a straightforward and measurable relationship between TV broadcasts and online traffic on company websites. Moreover, any change in life can affect people’s behavior, making separating ads’ effects on them almost impossible. Our framework represents a pioneering effort in quantifying the immediate impact of TV ads, with extensive experimental validation.

## 6. Conclusions

In the field of social sciences, the assessment of the efficiency of an ad has been predominantly examined through the perspective of marketing dynamics and consumer behavior. The most cited studies explored the ramifications of TV ads on aspects such as alcohol consumption patterns, dietary preferences in early childhood, eating habits, brand development, and audience perceptions. In fact, our study has introduced TV-Impact, a novel framework using machine learning techniques to quantitatively evaluate the immediate effect of TV ads on concurrent online traffic for the advertised brand. This framework, in particular, detects the online traffic of the advertising company right after broadcasting the ad.

Quantifying such an effect is tough because we cannot easily measure many factors which influence what people do, like current trends or news events. To deal with it, we used a method called CausalImpact, previously proposed by Google, which relies on Bayesian time-series learning. This method compares what happens to certain variables that should not be affected by the ad with what actually happens after the ad is shown on TV. It finds the effect by detecting the statistical difference between these control variables and the actual time-series signal after the event occurrence. The success of the model directly depends on the selected control variables. Our proposed TV-Impact framework enables choosing the most efficient control variables via a dynamic algorithm, using data from other companies’ ads and website visits. TV-Impact was tested with data from iLab, a Turkish investment company with 11 subsidiaries, and managed its companies’ ad strategies. This allowed us to accurately isolate and measure each company’s ad impact.

Secondly, we aimed to assess the immediate impact of ads on online traffic but encountered a challenge when ads from different companies broadcast simultaneously, creating interference in our data. To address this, we introduced a concept called ’Group Ad’. The Group Ad describes multiple ads broadcast in close succession, each with effects that are not immediately separable. We developed a supervised learning-based approach using the Random Forest algorithm to isolate the impact of these Group Ads and individual ads. It is capable of separating these combined effects. This approach, implemented within our TV-Impact framework, effectively distinguishes the impacts of both single and overlapped advertisements.

The third challenge arose in evaluating the success of TV-Impact, as there was no existing framework to compare it against. Furthermore, the continuous nature of ad campaigns causes one ad’s impact to mix with another’s. Hence, we focused on creating a structural model to identify and measure ad impacts. For assessment, we compared the results obtained with the TV-Impact with those obtained in the corresponding months of the prior year when our framework had not been applied. We examined the cost-efficiency of ads by comparing the budget per online session before and after implementing TV-Impact, based on a recommendation from the marketing department. This comparison showed that TV-Impact helped reduce spending per session.

In this study, we introduced TV-Impact, a novel framework designed to objectively quantify TV ads’ elusive and immediate effects on online web traffic, yielding a significant advancement in the field. To our knowledge, the pioneering framework captures the short-term consequences of TV ads on online Web channels. The framework’s performance was evaluated by comparing data from similar time periods with and without its implementation. Future research could refine this evaluation method, explore the long-term effects of advertising, and investigate the decrease in ad impact over time, which is crucial for optimizing the timing of ad campaigns. An advertisement impacts the consumer as soon as it is watched or in a very short time. Measuring the long-term effects may not be meaningful as it is generally contrary to the nature of advertisements. Still, it could provide an alternative to measuring events like COVID-19 or other prolonged incidents.

Another future perspective could be investigating the fading effects of advertising over time. This challenge is at least as challenging as measuring the immediate impact of advertising. However, developing a model that can explain the fading situation in ad effectiveness over time could greatly benefit advertising strategies, particularly in scheduling subsequent ad broadcasts.

## Figures and Tables

**Table 1:** Sample of online traffic logs collected from a company website. Logs are collected as number of sessions in four categories: direct, paid, organic, and referral.

Time	Direct Sessions	Paid Sessions	Organic Sessions	Referral Sessions
2 September 2023 00:00:07 UTC	0.0	1.0	1.0	0.0
2 September 2023 00:00:10 UTC	2.0	1.0	0.0	0.0
2 September 2023 00:00:26 UTC	1.0	0.0	1.0	0.0

**Table 2:** Enriched statistical data for a sample company.

Time	Direct Sessions	Direct Mean Last 7	Direct Median Last 7	Direct Q1 Last 7	Direct Q3 Last 7	Direct Mean Last 15	Direct Median Last 15	Direct Q1 Last 15	...	Organic Q3 Last 60
2 September 2023 00:00:00 UTC	2.0	−1.0	−1.0	−1.0	−1.0	0.4	1.0	0.0	...	9.0
2 September 2023 00:00:10 UTC	0.0	−1.0	−1.0	−1.0	−1.0	0.66	1.5	0.0	...	9.0
2 September 2023 00:00:20 UTC	10.0	−1.0	−1.0	−1.0	−1.0	1.0	1.0	1.0	...	8.0

**Table 3:** TV-Ad Data of a sample company. Data consists of TV ads’ characteristics such as broadcast time and duration of the ad.

Time	Channel	Channel Type	Prime Status	Measured/Non-Measured	Duration (Seconds)	Segment	Program
2 September 2023 00:00:11	BEIN SERIES 1	TEMATIK	PT	NM	15	SAGLIK	QUANTUM LEAP
2 September 2023 00:05:48	TEVE 2	OLCULEN	PT	M	15	KASKO	KANIT
2 September 2023 00:06:02	DMAX TV	OLCULEN	PT	M	10	SAGLIK	KONTEYNER SAVASLARI

**Table 4:** Average TV Ad distribution statistics for different *t* values.

*t*	Avg. Individual Ad Count	Avg. Group Ad Count	Avg. Number of Ads per Group Ad
2	86.07	70.02	3.15
3	54.87	66.60	3.76
4	38.20	56.13	4.77
6	22.33	42.00	6.82

**Table 5:** RMSE and MAE scores of Imaginary Ad post-period predictions for different *t* Values.

	Company 1		Company 2		Company 3	
Metric	RMSE	MAE	RMSE	MAE	RMSE	MAE
*t*						
2	8.91	7.04	10.09	8.69	7.79	6.16
3	12.00	9.79	8.89	7.10	7.77	6.01
4	8.84	7.02	11.87	7.62	6.95	5.55
6	9.41	7.78	7.82	6.20	6.32	5.09

**Table 6:** RMSE scores of Imaginary Ad post-period predictions for different thr and limit values.

	Company 1			Company 2			Company 3		
*thr*	0.4	0.5	0.6	0.4	0.5	0.6	0.4	0.5	0.6
*limit*									
3	6.45	6.32	6.59	7.45	7.36	7.67	7.87	7.47	5.76
5	6.54	6.19	6.56	8.08	7.64	7.82	8.32	7.53	5.71
10	7.57	6.28	6.58	8.65	7.73	7.78	9.00	7.61	5.83
15	8.61	6.51	7.26	9.67	7.79	7.78	9.28	7.54	6.21

**Table 7:** Yearly change in number of sessions and spending per ad for three companies.

Company	Change in Number of Session	Change in Spending per Ad
Company 1	+41.45%	−42.59%
Company 2	−11.10%	−69.74%
Company 3	−23.03%	−46.21%

## Data Availability

Data are contained within the article.
